# Asymmetric synthesis of biaryl atropisomers by dynamic resolution on condensation of biaryl aldehydes with (−)-ephedrine or a proline-derived diamine

**DOI:** 10.3762/bjoc.4.47

**Published:** 2008-12-04

**Authors:** Ann Bracegirdle, Jonathan Clayden, Lai Wah Lai

**Affiliations:** 1 School of Chemistry, University of Manchester, Oxford Rd., Manchester M13 9PL, UK

**Keywords:** atropisomer, biaryl, dynamic resolution, ephedrine, imdazolidine, oxazolidine

## Abstract

Atropisomeric biaryl aldehydes undergo diastereoselective condensation with (−)-ephedrine and with a proline-derived diamine, with selectivity highly dependent on solvent, temperature and reaction conditions. Levels of thermodynamic control up to 5:1 may be obtained by heating the diamine with the aldehyde in a sealed tube. Alternatively, crystallisation-induced dynamic transformation allows isolation of a single diastereoisomer in up to 85% yield. Hydrolysis and reduction of the major diastereoisomeric product of the reaction yields atropisomeric biaryls in >99:1 enantiomeric ratios.

## Introduction

Atropisomeric biaryl compounds have proved to be among the most successful of all chiral ligands for metal-catalysed asymmetric transformations [1–2]. Many biaryl ligands have been obtained in enantiomerically pure form by means of resolution [3], but there are also a number of important enantioselective methods for the synthesis of biaryls [4–9].

In view of the thermal instability inherent in a stereogenic bond (rather than a centre) dynamic methods appear particularly suited to the stereoselective synthesis of atropisomers [10–11]. In connection with our work on non-biaryl atropisomers such as amides [12–16], ethers [17] and ureas [18–20], we have explored the opportunities offered by dynamic kinetic [21–23] and dynamic thermodynamic [24] resolution [11,16,25–30]. We reported methods for the latter based on resolving “auxiliaries” which include silylethyl groups [28], proline-derived imidazolidines [25,27], ephedrine-derived oxazolidines [26–27], and, most extensively, sulfoxides [16,29–31]. These perform well when a powerful electronic or steric bias is evident about the atropisomeric bond over which control is applied [32], and in the case of atropisomeric amides have offered levels of conformational control up to 200:1 [33].

In this paper we present our extension of this work to the more conventional family of biphenyl compounds, which present a more challenging group of substrates because of the lack of steric or electronic contrast between the two conformers about an Ar–Ar bond. We find that a thermodynamic resolution is possible in certain cases and under rather precisely defined conditions. We propose a rationale for the selectivities observed which invoke thermodynamic resolution enabled by the presence of water.

## Results and Discussion

### Synthesis of the racemic substrates

**Scheme 1:**
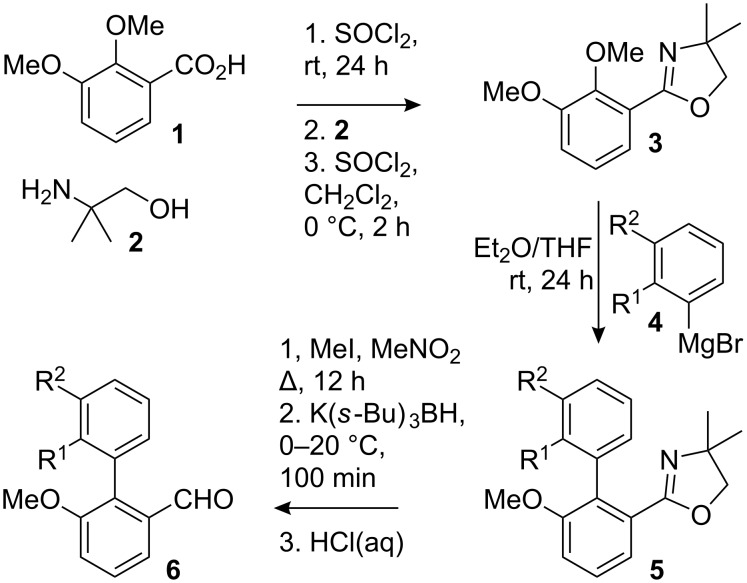
Synthesis of racemic aldehydes.

Previous success with stereocontrol employing ephedrine-derived oxazolidines [15,26–27,34–35] and proline-derived imidazolidines [25,27] prompted us to investigate the thermal stability and conformational preferences of similar products arising from condensation reactions of 2-formylbiaryls. A family of starting aldehydes **6a**–**f** was made by the method of Meyers [36]. 2,3-Dimethoxybenzoic acid (**1**) was converted via its acyl chloride to oxazoline **3**, from which the 2-methoxy group was displaced with a series of aryl Grignard reagents **4**, yielding biaryloxazolines **5a**–**f** (Scheme 1 and Table 1). Removal of the oxazoline by methylation, reduction and hydrolysis returned the aldehydes **6a**–**f**.

**Table 1:** Synthesis of biaryl aldehydes **6**.

entry	R^1^, R^2^	Oxazoline **5**, yield	Aldehyde **6**, yield

1	MeO, H	**5a**, 82	**6a**, 70
2	EtO, H	**5b**, 84	**6b**, 71
3	*i*-PrO, H	**5c**, 79	**6c**, 45
4	Me, H	**5d**, 80	**6d**, 71
5	Et, H	**5e**, 81	**6e**, 48
6	*i*-Pr, H	**5f**, 80	**6f**, 44
7	benzo^a^	**5g**, 91	**6g**, 31

^a^**4g** = 1-naphthylmagnesium bromide.

### Screening for selectivity by NMR

**Scheme 2:**
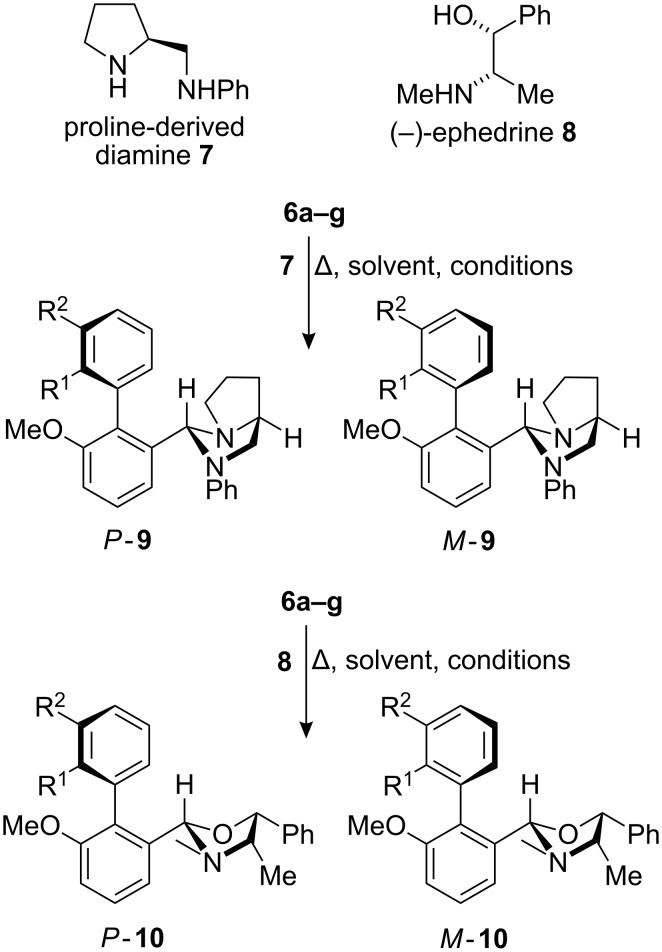
Diastereoisomeric imidazolidines and oxazolidines from biaryl aldehydes.

In order to investigate the ratios of diastereoisomers formed as these aldehydes condensed with the resolving agents, **6a** was mixed with 1 equiv of either **7** or **8** in toluene-*d*_8_ or benzene-*d*_6_ in an NMR tube (Scheme 2). The temperature of the tube was raised stepwise from rt to 110 °C as indicated in Table 2, allowing 30 min at each temperature and monitoring the changing ratio of diastereoisomers by ^1^H NMR. We assume that the condensations are diastereoselective at the new stereogenic centre, in accordance with literature precedent [15,25–27,34–35].

**Table 2:** Monitoring diastereoselectivity in the formation and/or interconversion of the diastereoisomers of imidazolidines **9a** and oxazolidines **10a**.

T/°C	*P*-**9a**_ _: *M*-**9a**^a,b^	*P*-**9a**_ _: *M*-**9a**^a,c^	*P*-**10a**_ _: *M*-**10a**^a,b^	*P*-**10a**_ _: *M*-**10a**^a,c^

25	1:1	1:1	–	–
40	1.5:1	1:1	–	–
50	1.5:1	1:1	1.7:1	1:1
60	1.5:1	1:1	1.5:1	1:1
70	1.5:1	–	1.2:1	1.2:1
80	1.8:1	1.6:1	–	1.2:1
90	5:1	1.8:1	–	1:1
100	5:1	–	2:1	–
110	5:1	–	2:1	–

^a^Stereochemistry assigned by X-ray crystallography: see below. ^b^Solvent = toluene-*d*_8_. ^c^Solvent = benzene-*d*_6_.

At temperatures below 70 °C, mixtures of diastereoisomeric imidazolidines **9** and oxazolidines **10** were observed with rather poor and somewhat variable selectivity (1:1–1.5:1). However, as the temperature rose above 80 °C in toluene-*d*_8_, the ratio of diastereoisomers of imidazolidines **9** also rose as high as 5:1. The ratio of oxazolidines **10** reached 2:1 under the same conditions. The ratios in benzene-*d*_6_ were somewhat lower in each case.

The increase in selectivity as the temperature rises is presumably the result of a thermodynamically controlled interconversion of the atropisomeric diastereoisomers of **9** and **10**. It seems probable however that the varying selectivities at lower temperatures are the result of a complex interplay between the kinetic and thermodynamic factors.

### Preparative dynamic resolutions

We decided to pursue this lead, and repeated the synthesis of **9** and **10** from **6a** and also the other aldehydes **6d** and **6g**. Equimolar quantities of **6** and either **7** or **8** were heated under a Dean-Stark condenser at reflux in either benzene, toluene or xylenes. The results are shown in Table 3.

**Table 3:** Preparative scale synthesis of imidazolidines **9** and oxazolidines **10** using (a) a Dean-Stark trap (DS) and (b) a sealed tube.

Starting aldehyde	**9**^a,b^ *P*:*M*	**9**^a,c^ *P*:*M*	**9**^a,d^ *P*:*M*	**10**^a,b^ *P*:*M*	**10**^a,c^ *P*:*M*	**10**^a,d^ *P*:*M*	**9**^c,e^ *P*:*M*	*P*-**9**/%^f^

**6a**	1:1	3:1^g,h^	1:1	1:1	3:1^g,i^	1:1	5:1^g^	27
**6b**	–	–	–	–	–	–	5:1^j^	37
**6c**	–	–	–	–	–	–	5:1^j^	34
**6d**	1:1	1:1	1:1	1:1	1:1	1:1	1:1	32
**6e**	–	–	–	–	–	–	1:1	24
**6f**	–	–	–	–	–	–	1:1	24
**6g**	1:1	1:1	1:1	1:1	1:1	1:1	1:1	27

^a^Carried out in a Dean Stark apparatus. ^b^Solvent = benzene. ^c^Solvent = toluene. ^d^Solvent = xylene. ^e^Carried out in a sealed tube.^ f^Yield after chromatographical purification. ^g^Stereochemistry assigned by X-ray crystallography: see below. ^h^81% isolated on crystallisation. ^i^85% isolated on crystallisation. ^j^Stereochemistry assigned by analogy with **10a**.

**Figure 1:**
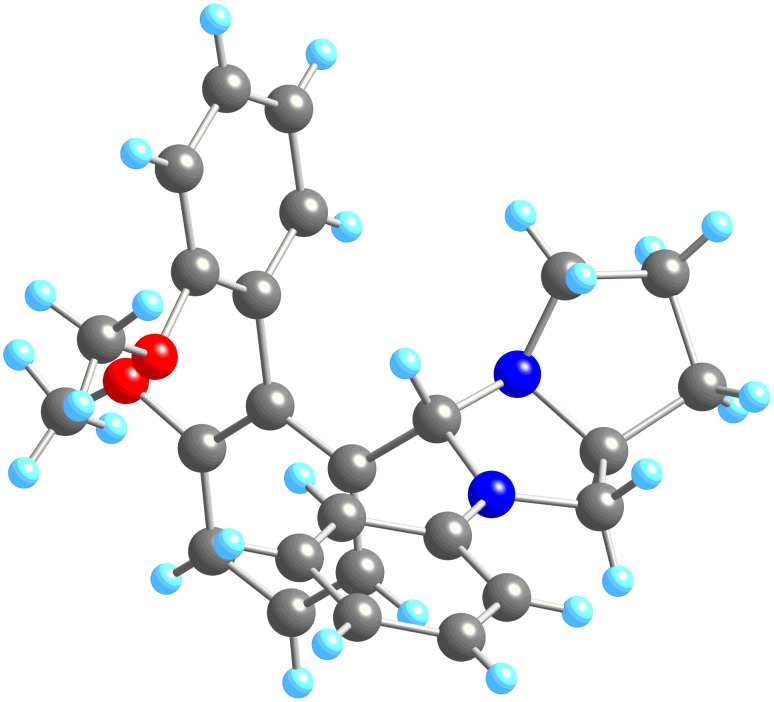
X-ray crystal structure of **9a**. X-ray data has been deposited with the Cambridge Crystallographic Data Centre: deposition number 693530.

**Figure 2:**
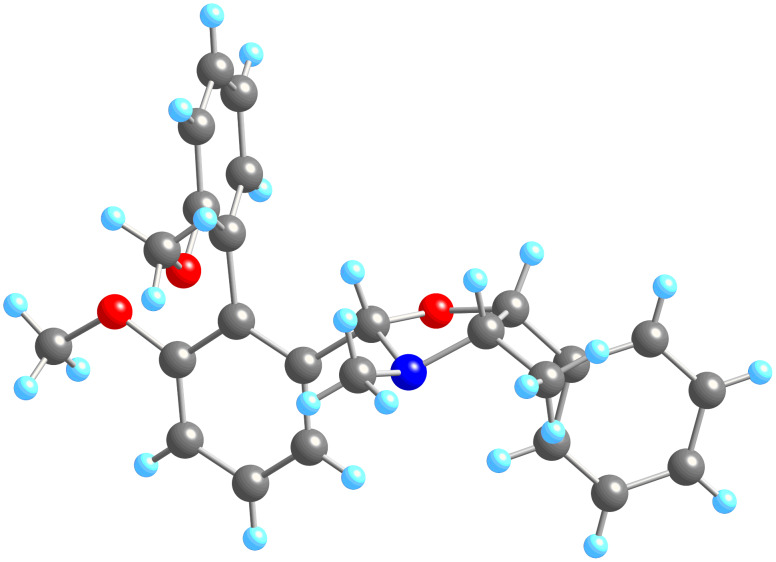
X-ray crystal structure of **10a**. X-ray data has been deposited with the Cambridge Crystallographic Data Centre: deposition number 693529.

Disappointingly, a ratio no greater than 3:1 was achieved, and this only when the reaction was conducted with **6a**. Moreover, the selectivity at both lower and higher temperatures was diminished to 1:1, a feature which suggests that toluene has some special feature as a solvent irrespective of its boiling temperature. Nonetheless, when solutions of **9a** and **10a** were cooled to room temperature, the major diastereoisomer in each case crystallised from the solution in good yield. The X-ray crystal structures of these two compounds are shown in Figure 1 and Figure 2. *P*-**9a** was obtained in 81% yield and *P*-**10a** in 85% yield, despite the major diastereoisomer making up only 75% of the crude reaction mixture as judged by NMR. The fact that the yield is greater than the selectivity in solution must represent a crystallisation-induced transformation of one diastereoisomer into the other.

Other than scale, there is one significant difference between the reactions carried out in the NMR tubes and those in the Dean-Stark apparatus: in the NMR experiments, water was not removed during the reaction, and it seemed possible that the continued presence of water in the reaction mixture was contributing to the higher selectivities observed under some conditions in Table 2.

The preparative results were therefore repeated by heating equimolar amounts of the diamine **7** and the aldehydes **6** in toluene in a sealed tube at 110 °C for 16 h. Pleasingly, in three cases, the same 5:1 selectivity was observed as in the NMR experiments. Isolated yields of the three major diastereoisomers were moderate due o the challenging nature of the chromatographic purification.

In order to exploit the asymmetric transformation of (±)-**6** into diastereoisomerically enriched *P*-**9**, a method for removal of the auxiliary was required. Previous experience [25,27] had shown that hydrolysis and *in situ* reduction allows isolation of related compounds bearing hydroxymethyl groups, whose barriers to racemisation are somewhat higher than those of the corresponding aldehydes [37] obtained by hydrolysis alone.

**Scheme 3:**
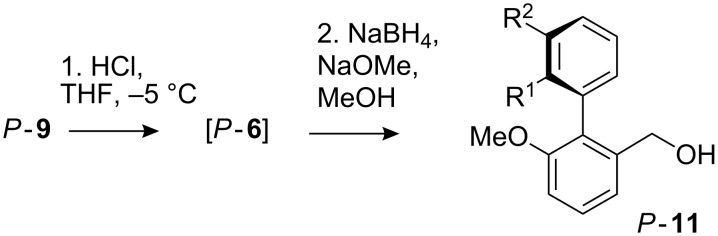
Atropisomeric alcohols by hydrolysis and reduction.

After trial reactions to establish optimal conditions, purified imidazolidines **9a** were treated with aqueous HCl in THF at −5 °C. After 35 min, a mixture of NaBH_4_ and NaOMe in methanol was added to neutralise the reaction mixture and to reduce the aldehyde to the atropisomeric alcohols **11** (Scheme 3). Table 4 shows the isolated yields of the essentially enantiomerically pure alcohols *P*-**11** obtained. Enantiomeric ratios were determined by ^1^H NMR in the presence of (+)-trifluoro-9-anthrylethanol, (+)-TFAE [38], comparing with authentic racemic samples of the alcohols made by simple reduction of (±)-**6**.

**Table 4:** Isolation of imidazolidines and hydrolysis/reduction to atropisomeric alcohols **11**.

Imidazolidine	alcohol	yield	Er^a^

*P*-**10a**	*P*-**11a**	55	>98:2
*P*-**10c**	*P*-**11c**	28	>98:2
*P*-**10d**	*P*-**11d**	44	>98:2
*P*-**10e**	*P*-**11e**	43	>98:2

^a^er determined by ^1^H NMR in the presence of (+)-TFAE [38].

### Rationalisations of results

**Scheme 4:**
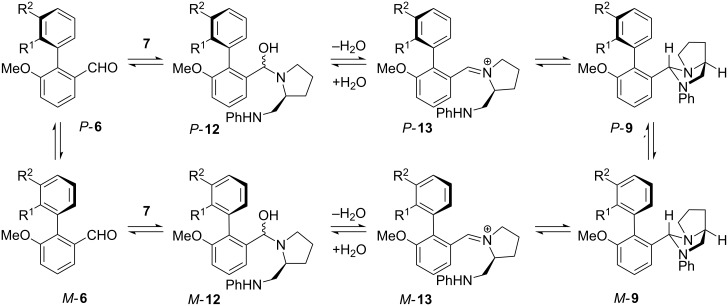
Mechanistic rationalisation of the dynamic resolution in the formation of **9**.

The improved selectivity observed on use of a sealed tube suggests that the presence of water plays an important role in the determining the selectivity of the reaction (Scheme 4). The ratio of isomers of **9** observed in the NMR tube slowly improved on raising the temperature, and we propose that this observation is consistent with selectivity being under thermodynamic control, with *P*-**9** being more stable than *M*-**9**. However it seems that the attainment of the favourable equilibrium mixture of diastereoisomers requires both heat (to allow rotation about the hindered Ar–Ar bond in **6** or one of its derivatives) and water. Water would allow the imidazolidines **9** to hydrolyse back via iminiums **13** to hemiaminals **12** or the starting aldehydes **6**, which presumably have a lower barrier to bond rotation [37]. These observations are therefore consistent with the following rationalisation: in toluene, *P*-**6** and *M*-**6** may interconvert, and both react with diamine **7** to yield hemiaminals **12** and hence imidazolidines **9**. Kinetic selectivity (such as that observed at lower temperatures, for example in benzene) is low. Moreover, *P*-**9** and *M*-**9** do not interconvert directly by bond rotation even in refluxing xylenes (we have confirmed this by heating *P*-**9a** under these conditions). Nonetheless, the two diastereoisomers may attain thermodynamic equilibrium if an alternative mechanism for their interconversion presents itself, namely hydrolysis back to the aldehyde **6** (or maybe the hemiaminal **12**). *P*-**9** is more stable than *M*-**9**, and equilibration allows the ratio of *P:M*-**9** to build up to about 5:1. High selectivity is disrupted if (a) the temperature is too low (presumably the case in reactions carried out in benzene: see Table 2) or (b) water is driven out of the reaction either by high temperatures or by the use of a Dean-Stark apparatus. Toluene at 110 °C provides the right balance of boiling point with ability to retain in solution a sufficient concentration of water to allow equilibration via the starting aldehydes.

## Conclusion

Biaryl aldehydes may be resolved in a dynamic fashion by condensation with (−)-ephedrine, or, more effectively, a proline-derived diamine. The selectivity of the dynamic resolution depends significantly on conditions of the reaction, and is the result of a complex interplay of kinetic and thermodynamic effects. The best selectivities, of up to 5:1, were obtained on reaction of alkoxy-substituted biaryls with the diamine in a sealed tube, presumably because water plays a role in assisting interconversion of the atropisomeric products and allows thermodynamic equilibrium to be attained. The imidazolidine products may be hydrolysed and reduced to atropisomeric alcohols in moderate yield and with excellent enantioselectivity.

## Experimental

For general experimental procedures, see Supporting Information File 1.

### General procedure for formation of imidazolidines **9**

The aldehyde **6** (1 mmol) was heated at 110 °C with (*S*)-(+)-2-(anilinomethyl)pyrrolidine [39] (1 mmol) in toluene (25 mL) in a sealed tube overnight. The solvent was evaporated under reduced pressure, and the product was purified by flash chromatography to yield the corresponding imidazolidine.

#### (3*R*,7a*S*)-3-(6,2′-Dimethoxybiphenyl-2-yl)-2-phenylhexahydropyrrolo[1,2-*c*]imidazole (**9a**)

In this way, but on a 2 mmol scale, aldehyde **6a** gave, after purification by flash chromatography on alumina (eluent 5:1 v/v petroleum ether/EtOAc), the title compound **9a** as a white solid (680 mg, 81%). Mp 161–164 °C; ^1^H NMR spectra indicated a mixture of conformers at a ratio of 5:1. Extensive purification by flash chromatography on alumina (eluent 5:1 v/v petroleum ether/EtOAc) resulted in the separation of one diastereoisomer (227 mg, 33% of initial yield). Mp 170–175 °C; R_f_  0.32 (5:1 v/v petroleum ether/EtOAc ); IR ν_max_ (thin film) (DCM) 3012, 2933, 1637, 1599, 1050, 1434 cm^−1^; ^1^H NMR (300 MHz, CDCl_3_) δ; 7.44–7.38 (1H, m, biaryl-*H*), 7.38–7.33 (1H, m, biaryl-*H*), 7.28 (1H, t, *J* 8, biaryl-*H*), 7.19 (2H, dt, *J* 7 and 1, biaryl-*H*), 7.12 (1H, dt, *J* 6 and 1, biaryl-*H*), 7.04 (1H, d, *J* 8, Ph-*H*), 6.96 (1H, *J* 8, Ph-*H*), 6.68 (1H, t, *J* 7, Ph-*H*), 6.58 (2H, d, *J* 8, Ph-*H*), 5.02 (1H, s, C*H*N), 3.92 (3H, s, OC*H*_3_), 3.85–3.72 (1H, m, PhNCH_2_C*H*), 3.78 (3H, s, OC*H*_3_), 3.17 (1H, t, *J* 9, PhNC*H*_2_), 2.55–2.46 (1H, m, PhNC*H*_2_), 2.28–2.17 (1H, m, NC*H*_2_), 2.15–2.02 (1H, m, NC*H*_2_), 1.87–1.65 (4H, m, NCH_2_C*H*_2_C*H*_2_); ^13^C NMR (75 MHz, CDCl_3_) δ; 157.5 (MeOC), 156.2 (MeOC), 146.0 (N*C*[C_5_H_5_]), 142.5 (*C*-CHN), 133.1, 128.8, 128.5, 128.4 (aromatics), 126.2, 125.2 (Cq), 120.3, 117.2, 115.8, 112.4, 110.4, 110.2 (aromatics), 81.5^+^ (CHN), 60.3^+^ (N*C*HCH_2_), 56.0^+^ (OMe), 55.2^+^ (OMe), 53.2^−^ (CH_2_), 53.2^−^ (CH_2_); *m/z* (CI) 401 (M+1, 100%). Mass measurement 400.2143, C_26_H_28_N_2_O_2_ requires 400.2150. [α]_D_^25^ = +213.2 (*c* = 0.201, ethanol).

Also obtained was a mixture of both diastereoisomers (453 mg, 67% of initial yield).

In another experiment, the crude mixture was recrystallised from IPA to yield **9a** as a crystalline solid (81%).

### General procedure for formation of oxazolidines **10**

The aldehyde **6** (1 mmol) was heated to reflux with (1*R*,2*S*)-(−)-ephedrine (1 mmol) in toluene (25 mL) for 24 h. The solvent was evaporated under reduced pressure, and the product **10** was purified by flash chromatography to yield the corresponding oxazolidine.

#### (2*S*,4*S*,5*R*)-2-(6,2′-Dimethoxybiphenyl-2-yl)-3,4-dimethyl-5-phenyloxazolidine (**10a**)

Aldehyde **6a** (608 mg, 2.51 mmol) gave, after flash chromatography on alumina (eluent 5:1 v/v petroleum ether/EtOAc), the title compound **10a** as a white solid (731 mg, 75%). Mp 131–135 °C; ^1^H NMR spectra indicated a mixture of conformers at a ratio of 3:1.

In another experiment, recrystallisation of the crude material from IPA gave *(P)*-**10a** as a crystalline solid (85%). R_f_ 0.40 (5:1 v/v petroleum ether/EtOAc ); IR ν_max_ (thin film) (DCM) 3124, 2954, 1582, 1499, 1250, 801 cm^−1^; ^1^H NMR (300 MHz, CDCl_3_) δ; 7.80−7.02 (12H, m, Ar*H*), 5.05 (1H, d, *J* 8, PhC*H*), 4.40 (1H, s, NC*H*O), 3.84 (6H, s, OC*H*_3_), 2.85 (1H, qn, *J* 7, CH_3_C*H*), 2.15 (3H, s, NC*H*_3_), 0.80 (3H, d, *J* 7, CHC*H*_3_); ^13^C NMR (75 MHz, CDCl_3_): 164.8, 164.2, 139.3, 136.2, 129.8, 128.5, 128.2, 128.1, 127.6, 127.1, 126.5, 126.2, 125.9, 124.3, 122.3, 122.1, 114.3, 113.9, 93.6, 82.1, 65.4, 57.2, 56.5, 37.6, 14.5; *m/z* (CI) 390 (M+1, 100%). Mass measurement 389.1985, C_25_H_27_NO_3_ requires 389.1991. Anal. Calcd for C_25_H_27_NO_3_: C, 77.09, H, 6.99, N, 3.60; Found C, 76.79; H, 7.13; N, 3.66. [α]_D_^25^ −134.9 (*c* = 0.512, chloroform).

## Supporting Information

Procedures for the synthesis, and characterisation data, of the remaining compounds reported in this paper.

File 1 Full experimental data for all new compounds reported in the paper.
