# Supplementary material for: Asymmetric synthesis of biaryl atropisomers by dynamic resolution on condensation of biaryl aldehydes with (−)-ephedrine or a proline-derived diamine
Source: Beilstein J Org Chem. 2008 Dec 4;4:47. doi: 10.3762/bjoc.4.47 (PMC2633665; doi:10.3762/bjoc.4.47)
Supplement: File 1 — Full experimental data for all new compounds reported in the paper. [file Beilstein_J_Org_Chem-04-47-s001.doc]

**Asymmetric synthesis of biaryl atropisomers by dynamic resolution during condensation of biaryl aldehydes with (**−**)-ephedrine or a proline-derived diamine**

Ann Bracegirdle, Jonathan Clayden* and Lai Wah Lai

Address: School of Chemistry, University of Manchester, Oxford Rd., Manchester M13 9PL, UK

* Corresponding author

**SUPPORTING INFORMATION**

# General Information

NMR spectra were recorded on a Varian XL 300 or a Bruker Ultrashield 300, 400 or 500 spectrometer. The chemical shifts (δ) are reported in ppm downfield of trimethylsilane and coupling constants (*J*) reported in Hz and rounded to 0.5 Hz. Splitting patterns are abbreviated as follows: singlet (s), doublet (d), triplet (t), quartet (q), mulitiplet (m), broad (br), or a combination of these.

EI and CI spectra were recorded on a Fisons VG Trio 2000; and high resolution mass spectra (HRMS) were recorded on a Kratos Concept-IS mass spectrometer, and are accurate to ± 0.001. Infrared spectra were recorded on an Ati Matson Genesis Series FTIR spectrometer as a film on a sodium chloride plate. Absorptions reported are sharp and strong unless otherwise stated as broad (br), medium (m), or weak (w), only absorption maxima of interest are reported. Melting points (mp) were determined on a Gallenkamp apparatus and are uncorrected.

Thin layer chromatography (TLC) was performed using commercially available pre-coated plates (Macherey-Nagel alugram Sil G/_UV254_) and visualised with UV light at 254 nm or phosphomolybdic acid dip (5% in ethanol). Flash chromatography was carried out using Fluorochem Davisil 40–63 μ 60 Å.

*2-(2,3-Dimethoxyphenyl)-4,4-dimethyl-4,5-dihydrooxazole* (**3**)

2,3-Dimethoxybenzoic acid (12.2 g, 67 mmol) was cooled to 0 °C, and thionyl chloride (15 mL, 3 equiv) was added dropwise. The resulting mixture was stirred at 0 °C for 1 h and then warmed to r. t. and stirred for a further 24 h. Excess thionyl chloride was removed under reduced pressure, yielding the acid chloride as a brown solid. The acid chloride (15.6 g, 77.61 mmol) was dissolved in DCM (60 mL) and cooled to 0 °C. 2-Amino-2-methyl-propanol (15.22 g, 2.2 equiv) was dissolved in DCM (50 mL) and added dropwise to the mixture, which was then warmed to r. t. and stirred for 3 h. A white precipitate formed (hydrochloride of 2-amino-2-methyl-1-propanol) which was removed by filtration. The solution was concentrated under reduced pressure, then dissolved in DCM (100 mL) and cooled to 0 °C. Thionyl chloride (19.2 mL, 3 equiv) was added drop wise, and the mixture was warmed to r. t. and stirred at r. t. for 3 h. The mixture was cooled to 0 °C, and water (50 mL) was added drop wise, followed by 40% NaOH (50 mL). The organic layer was extracted, washed with brine, dried and concentrated under reduced pressure. The resultant yellow oil was purified by distillation (kugelrohr) to yield the title compound **3** as a pale yellow solid (11.5 g, 73%). Mp 48–49 °C (lit. [1] 44–46 °C); *R_f_* 0.35 (10:1 v/v petroleum ether/EtOAc); IR ν_max_ (thin film) (DCM) 2843, 1470, 1383, 1153, 735 cm^-1^; ^1^H NMR (300 MHz, CDCl_3_) δ; 6.92–7.5 (3H, m, Ar*H*), 4.13 (2H, s, OC*H_2_*C(CH_3_)_2_N), 3.82 (3H, s, OC*H_3_*), 3.80 (3H, s, OC*H_3_*), 1.38 (3H, s, C*H_3_*), 1.35 (3H, s, C*H_3_*); ^13^C NMR (75 MHz, CDCl_3_) δ; 161.1, 147.3, 146.5, 123.7, 123.2, 122.7, 115.1, 79.0, 69.5, 61.2, 55.3, 24.2; *m/z* (CI) 236 (M+1, 100%). Mass measurement 235.1208, C_13_H_17_NO_3_ requires 235.1201.

**General procedure for formation of biaryl oxazolines 5.**

Magnesium turnings (2.82 g) in anhydrous diethyl ether (100 mL) were stirred under N_2_. 1,2-Dibromoethane (2 drops) was added followed by the appropriate 2-bromoaryl (3 equiv) in anhydrous diethyl ether (75 mL) and the mixture was stirred at r. t. for 2 h. A solution of oxazoline **3** (27.66 mmol) in anhydrous THF (150 mL) was added drop wise. The solution was stirred at r. t. for 24 h, or until the reaction was complete by TLC (petroleum ether/ethyl acetate, 7:3). The reaction was quenched by dropwise addition of saturated ammonium chloride (50 mL) followed by water (50 mL). The product was extracted with diethyl ether (3 x 100 mL), the combined organic layers were dried with magnesium sulphate, concentrated under reduced pressure, and purified by recrystallisation using EtOAc/Hexane (1:1).

*2-(6,2’-Dimethoxybiphenyl-2-yl)-4,4-dimethyl-4,5-dihydrooxazole* (**5a**)

By this method, 2-bromoanisole (10.23 mL, 3 equiv) gave the title compound **5a** as a pale yellow solid (7.1 g, 82%). Mp 130–131 °C (lit. [1] 127–129 °C); *R_f_* 0.20 (10:1 v/v petroleum ether/EtOAc); IR ν_max_ (thin film) (DCM) 3331(b), 2965, 1657, 1465, 1352, 1129, 745 cm^-1^; ^1^H NMR (300 MHz, CDCl_3_) δ; 6.82–7.30 (7H, m, Ar*H*), 3.80 (6H, s, OC*H_3_*) 3.6 (1H, d, *J* 8), 3.55 (1H, d, *J* 8), 1.15 (6H, s, C*H_3_*); ^13^C NMR (75 MHz, CDCl_3_) δ; 165.3, 127.3, 127.1, 124.2, 123.1, 122.3, 121.2, 119.2, 118.9, 117.3, 116.8, 116.2, 115.3, 70.3 (*C*H_2_), 56.3 (*C*(CH_3_)_2_), 54.2 (O*C*H_3_), 23.1 (*C*H_3_); *m/z* (CI) 312 (M+1, 100%), Mass measurement 311.1519, C_19_H_21_NO_3_ requires 311.1513.

*2-Methoxy-2’-ethoxy-6-(4,4-dimethyloxazolin-2-yl)biphenyl* (**5b**)

In the same way 1-bromo-2-ethoxybenzene (16.7 g, 3 equiv) gave the title compound **5b** as a white solid (7.5 g, 84%). Mp 138–141 °C; *R_f_* 0.22 (10:1 v/v petroleum ether/EtOAc); IR ν_max_ (thin film) (DCM) 3050, 2985, 2762, 1623, 1501, 1321, 1050, 773 cm^-1^; ^1^H NMR (300 MHz, CDCl_3_) δ; 7.38 (1H, d, *J* 6, Ar*H*), 7.32 (1H, d, *J* 6, Ar*H*), 7.21 (1H, d, *J* 6, Ar*H*), 7.1–7.0 (3 H, m, ArH x 3), 6.94 (1H, t, *J* 6, Ar*H*), 4.06 (2H, q, *J* 7, ArOC*H_2_*CH_3_), 3.79 (3H, s, ArOC*H_3_*), 3.74 (1H, d, *J* 8, C*H_2_*), 3.64 (1H, d, *J* 8, C*H_2_*), 1.46 (3H, t, *J* 7, OCH_2_C*H_3_*), 1.23, (3H, s, (C*H_3_*), 1.20 (3H, s, CH_3_); ^13^C NMR (75 MHz, CDCl_3_) δ; 163.8, 159.2, 157.4, 131.3, 130.9, 128.8, 127.9, 127.7, 126.7, 121.9, 116.1, 114.7, 113.0, 79.5, 64.3, 63.9, 56.1, 28.3, 15.1, 15.0; *m/z* (CI) 326 (M+1, 70%), 343 (M+18, 100%). Mass measurement *M+H^+^* 326.1701, C_20_H_23_NO_3_ *M+H^+^* requires 326.1756.

*2-Methoxy-2’-isopropoxy-6-(4,4-dimethyloxazolin-2-yl)biphenyl* (**5c**)

In the same way 1-bromo-2-isopropoxybenzene (17.7 g, 3 equiv) gave the title compound **5c** as a pale yellow solid (7.4 g, 79%). Mp 155–157 °C; *R_f_* 0.25 (10:1 v/v petroleum ether/EtOAc); IR ν_max_ (thin film) (DCM) 2843, 1877, 1633, 1421, 1313, 1219, 1028, 745 cm^-1^; ^1^H NMR (CDCl_3_) δ; 7.42–7.24 (3H, m, Ar*H*), 7.21 (1H, d, *J* 7, Ar*H*), 7.07 (1H, d, *J* 7, Ar*H*), 7.00–6.94 (2H, m, Ar*H*), 4.37 (1H, sep, *J* 6, ArOC*H*(CH_3_)_2_), 3.78 (3H, s, ArOC*H_3_*), 3.72 (1H, d, *J* 8, C*H_2_*), 3.65 (1H, d, *J* 8, C*H_2_*), 1.40 (6H, d, *J* 7, OCH(C*H_3_*)*_2_*), 1.21 (3H, s, C*H_3_*), 1.18 (3H, s, CH_3_)*,*; ^13^C NMR (75 MHz, CDCl_3_) δ; 163.8, 157.4, 154.8, 132.6, 130.8, 129.7, 126.7, 122.2, 121.9, 120.3, 115.9, 114.0, 113.7, 79.5, 77.7, 69.9, 67.3, 56.2, 22.6, 22.5, 22.1; *m/z* (CI) 340 (M+1, 100%). Mass measurement 339.1838, C_21_H_25_NO_3_ requires 339.1834.

*2-(6-Methoxy-2’-methyl-biphenyl-2-yl)-4,4-dimethyl-4,5-dihydrooxazole* (**5d**)

In the same way 2-bromotoluene (3.6 mL, 3 equiv) gave the title compound **5d** as a white solid (7.1 g, 80%). Mp 125–126 °C; *R_f_* 0.25 (10:1 v/v petroleum ether/EtOAc); IR ν_max_ (thin film) (DCM) 3056, 2832, 1600, 1450, 1237, 753 cm^-1^; ^1^H NMR (300 MHz, CDCl_3_) δ; 7.0–7.40 (7H, m, Ar*H*), 3.79 (3H, s, OC*H_3_*), 3.70 (1H, d, *J* 8, C*H_2_*), 3.55 (1H, d, *J* 8, C*H_2_*), 2.15 (3H, s, Ar-C*H_3_*), 1.23 (3H, s, C*H_3_*), 1.20 (3H, s, C*H_3_*); ^13^C NMR (75 MHz, CDCl_3_) δ; 169.7, 163.1, 129.9, 129.2, 128.1, 127.4, 127.3, 127.1, 126.7, 126.6, 123.2, 117.0, 67.1 (*C*H_2_), 56.3, (*C*(CH_3_)_2_), 51.2 (O*C*H_3_), 23.0 (*C*H_3_); *m/z* (CI) 296 (M+1, 100%). Mass measurement 295.1565, C_19_H_21_NO_2_ requires 295.1572.

*2-Methoxy-2’-ethyl-6-(4,4-dimethyloxazolin-2-yl)biphenyl* (**5e**)

In the same way, 1-bromo-2-ethylbenzene (15.4 g, 3 equiv) gave the title compound **5e** as a white solid (6.9 g, 81%). Mp 133–134 °C; *R_f_* 0.24 (10:1 v/v petroleum ether/EtOAc); IR ν_max_ (thin film) (DCM) 3004, 2237, 1665, 1526, 1498, 1404, 1395, 1119, 1046, 777, 713 cm^-1^; ^1^H NMR (CDCl_3_) δ; 7.41–7.06 (7H, m, Ar*H*), 3.78 (3H, s, ArOC*H_3_*), 3.72 (1H, d, *J* 8, C*H_2_*), 3.56 (1H, d, *J* 8, C*H_2_*), 3.40 (2H, q, *J* 7, ArC*H_2_*CH_3_), 1.32 (3H, t, *J* 7, ArCH_2_C*H_3_*), 1.25, (6H, s, NC(C*H_3_*)*_2_*); ^13^C NMR (75 MHz, CDCl_3_) δ; 163.5, 156.9, 130.2, 129.8, 129.3, 128.7, 127.7, 127.5, 125.6, 125.2, 125.1, 121.7, 112.8, 79.6, 67.4, 56.0, 28.2, 25.9, 23.2, 21.8; *m/z* (CI) 310 (M+1, 80%). Mass measurement 309.1726, C_20_H_23_NO_2_ requires 309.1729.

*2-Methoxy-2’-isopropyl-6-(4,4-dimethyloxazolin-2-yl)biphenyl* (**5f**)

In the same way 1-bromo-2-isopropylbenzene (16.5 g, 3 equiv) gave the title compound **5f** as a white solid (7.1 g, 80%). Mp 142–143 °C; *R_f_* 0.18 (10:1 v/v petroleum ether/EtOAc); IR ν_max_ (thin film) (DCM) 2974, 2387, 1728, 1598, 1503, 1466, 1395, 1257, 1123, 1046, 923 cm^-1^; ^1^H NMR (300 MHz, CDCl_3_) δ; 7.38 (1H, d, *J* 8, Ar*H*), 7.36–7.12 (4H, m, Ar*H*), 7.09 (1H, d, *J* 7, Ar*H*), 7.06 (1H, d, *J* 7, Ar*H*), 3.77 (3H, s, ArOC*H_3_*), 3.70 (1H, d, *J* 9, C*H_2_*), 3.58 (1H, d, *J* 9, C*H_2_*), 2.71 (1H, sep, *J* 8, ArC*H*(CH_3_)_2_), 1.21, (3H, s, C*H_3_*), 1.18, (3H, s, C*H_3_*), 1.11 (6H, d, *J* 8, ArCH(C*H_3_*)*_2_*); ^13^C NMR (75 MHz, CDCl_3_) δ; 163.6, 157.1, 131.0, 130.1, 129.8, 128.6, 127.1, 127.5, 126.8, 125.9, 124.6, 121.7, 112.7, 79.5, 67.4, 56.0, 29.7, 28.2, 28.1, 14.7, 14.5; *m/z* (CI) 324 (M+1, 100%). Mass measurement 323.1883, C_21_H_25_NO_2_ requires 323.1885.

*2-[3-Methoxy-(2-naphthalen-1-yl)phenyl]-4,4-dimethyl-4,5-dihydrooxazole* (**5g**)

In the same way, 1-bromonaphthalene (4.2 mL, 3 equiv) gave the title compound **5g** as a white solid (3.6 g, 91%). Mp 148–150 °C; *R_f_* 0.25 (10:1 v/v petroleum ether/EtOAc); IR ν_max_ (thin film) (DCM) 2843, 1470, 1383, 1383, 1153, 735 cm^-1^; ^1^H NMR (300 MHz, CDCl_3_) δ; 7.0–7.65 (10H, m, Ar*H*), 3.60 (3H, s, OC*H_3_*), 3.22 (2H, AB m), 0.90 (3H, s, C*H_3_*), 0.85 (3H, s, C*H_3_*); ^13^C NMR (75 MHz, CDCl_3_) δ; 167.3, 155.9, 133.9, 133.6, 132.7, 131.7, 130.4, 130.2, 129.9, 129.7, 129.1, 125.9, 125.7, 123.5, 123.0, 122.7, 121.3, 67.2 (*C*H_2_), 57.9 (*C*(CH_3_)_2_), 56.3 (O*C*H_3_), 23.9 (*C*H_3_); *m/z* (CI) 332 (M+1, 100%), Mass measurement 331.1566, C_22_H_21_NO_2_ requires 331.1572.

# General procedure for formation of aldehydes 6.

The oxazoline **5** (8.6 mmol) was dissolved in nitromethane (50 mL). Iodomethane (2 equiv) was added and the mixture was heated to reflux for 12 h. The solvent was removed under reduced pressure to yield the methylated oxazoline. This material was suspended in THF (10 mL) at 0 °C and K-Selectride® (1 equiv) was added over 40 min. The yellow solution was warmed to RT and stirred for 1 h. The mixture was acidified with 5% HCl (aq) (10mL) and stirring was continued until TLC indicated total consumption of starting material (7:3 petroleum ether/EtOAc). The solvents were evaporated under reduced pressure, and the residue was dissolved in THF (5mL) and treated with 10% NaOH:30% H_2_O_2_ (2.5:1/5, 4 mL). The product was extracted with pentane (3 x 25 mL), washed with water (3 x 25 mL) and brine (3 x 25 mL), the combined organic layers were dried with magnesium sulfate and concentrated under reduced pressure. The resultant crude product was purified by flash chromatography.

*6,2’-Dimethoxybiphenyl-2-carbaldehyde* (**6a**)

By this method, oxazoline **5a** (8.9 mmol) gave, after purification by flash column chromatography (eluent: 7:3 v/v petroleum ether/EtOAc) the title compound **6a** as a white solid (1.5 g, 70%). Mp 66–68 °C (lit. [1] 65–67 °C); *R_f_* 0.75 (7:3 v/v petroleum ether/EtOAc); IR ν_max_ (thin film) (DCM) 3027, 2934, 2836, 1689, 1539, 735 cm^-1^; ^1^H NMR (300 MHz, CDCl_3_) δ; 9.75 (1H, s, C*H*O), 8.70 (1H, d, *J* 7.5, Ar*H*), 7.52–7.30 (4H, m, Ar*H*), 7.15 (1H, d, *J* 7 Ar*H*), 7.11 (1H, d, *J* 8, Ar*H*), 3.84 (3H, s, OC*H*_3_), 3.81 (3H, s, OC*H*_3_); ^13^C NMR (75 MHz, CDCl_3_) δ; 192 (*C*HO), 167.4, 162.1, 128.3, 128.1, 126.9, 126.3, 125.1, 124.7, 124.1, 123.6, 117.9, 113.5, 56.2 (O*C*H_3_), 55.1 (O*C*H_3_); *m/z* (CI) 243 (M+1, 100%). Mass measurement 242.0920, C_15_H_14_O_3_ requires 242.0943.

*6-Methoxy-2’-ethoxybiphenyl-2-carbaldehyde* (**6b**)

In the same way, oxazoline **5b** (7.9 mmol) gave the title compound **6b** as a white solid (1.4 g, 71%). Mp 72–74 °C; *R_f_* 0.80 (7:3 v/v petroleum ether/EtOAc); IR ν_max_ (thin film) (DCM) 3021, 2555, 1689 (C=O) 1585, 1411, 1393, 1301, 1211, 1198, 762; ^1^H NMR (300 MHz, CDCl_3_) δ; 9.74 (1H, s, C*H*O), 7.66 (1H, d, *J* 7, Ar*H*), 7.48 (1H, t, *J* 7, Ar*H*), 7.30–7.22 (3H, m, Ar*H*), 7.08 (1H, t, *J* 7, Ar*H*), 7.04 (1H, d, *J* 7, Ar*H*), 4.0 (2H, q, *J* 7, ArOC*H_2_*CH_3_), 3.81 (3H, s, OC*H*_3_), 1.22 (3H, t, *J* 7, ArOCH_2_C*H_3_*); ^13^C NMR (75 MHz, CDCl_3_) δ; 193.2, 157.4, 156.8, 135.5, 132.9, 131.7, 129.9, 128.8, 122.3, 120.5, 118.9, 116.4, 112.2, 64.1, 56.3, 14.8; *m/z* (CI) 257 (M+1, 60%), 274 (M+18, 100%). Mass measurement *M+NH_4_^+^* 274.1439, C_16_H_16_O_3_ *M+NH_4_^+^* requires 274.1443.

*6-Methoxy-2’-isopropoxybiphenyl-2-carbaldehyde* (**6c**)

In the same way, oxazoline **5c** (3.9 g, 11.5 mmol) gave the title compound **6c** as a white solid (1.4 g, 45%). Mp 78–80 °C; *R_f_* 0.75 (7:3 v/v petroleum ether/EtOAc); IR ν_max_ (thin film) (DCM) 2976, 2836, 1696, 1592, 1467, 1383, 1261, 1126; ^1^H NMR (300 MHz, CDCl_3_) δ; 9.74 (1H, s, C*H*O), 7.66 (1H, d, *J* 8, Ar*H*), 7.52–6.78 (6H, m, Ar*H*), 4.02 (1H, sep, *J* 8, ArOC*H*(CH_3_)_2_), 3.78 (3H, s, ArOC*H*_3_), 1.24 (6H, d, *J* 8 ArOCH(C*H_3_*)*_2_*); ^13^C NMR (75 MHz, CDCl_3_) δ; 194.8, 157.8, 157.2, 133.5, 132.9, 131.1, 130.9, 129.7, 124.3, 122.9, 117.8, 115.4, 113.9, 63.1, 59.4, 15.2, 15.0; *m/z* (CI) 271 (M+1, 80%), 288 (M+18, 100%). Mass measurement 270.1256, C_17_H_18_O_3_ requires 270.1263.

*6-Methoxy-2’-methylbiphenyl-2-carbaldehyde* (**6d**)

In the same way, oxazoline **5d** (4.73 mmol) gave **6d** as a white solid (750 mg, 71%). Mp 65–67 °C; *R_f_* 0.75 (7:3 v/v petroleum ether/EtOAc); IR ν_max_ (thin film) (DCM) 3013, 2836, 2913, 1685, 743 cm^-1^; ^1^H NMR (300 MHz, CDCl_3_) δ; 9.6 (1H, s, C*H*O), 7.60 (1H, d, *J* 7, Ar*H*), 7.4 (1H, t, *J* 8 Ar*H*), 7.22–7.30 (m, 4H, Ar*H*), 7.15 (1H, d, *J* 7.5, Ar*H*), 3.70 (3H, s, OC*H*_3_), 2.0 (3H, s, C*H*_3_); ^13^C NMR (75 MHz, CDCl_3_) δ; 183.6, 155.2, 137.3, 133.7, 133.1, 129.3, 128.6, 128.1, 127.9, 127.5, 127.3, 126.1, 125.3, 55.2 (O*C*H_3_), 12.1 (*C*H_3_); *m/z* (CI) 244 (M + 18, 100%), 227 (M+ 1, 75%). Mass measurement 226.0987, C_15_H_14_O_2_ requires 226.0994.

*6-Methoxy-2’-ethylbiphenyl-2-carbaldehyde* (**6e**)

In the same way, oxazoline **5e** (4.0 g, 12.9 mmol) gave the title compound **6e** as a white solid (1.5 g, 48%). Mp 68–69 °C; *R_f_* 0.80 (7:3 v/v petroleum ether/EtOAc); IR ν_max_ (thin film) (DCM) 3445, 2929, 2340, 1685 (C=O) 1592, 1466, 1383, 1260, 1125, 952 cm^-1^; ^1^H NMR (300 MHz, CDCl_3_) δ; 9.67 (1H, s, ArC*H*O), 7.68 (1H, d, *J* 8, Ar*H*), 7.52 (1H, t, *J* 8, Ar*H*), 7.43–7.27 (3H, m, Ar*H*), 7.23 (1H, d, *J* 8, Ar*H*), 7.15 (1H, d, *J* 8, Ar*H*), 3.81 (3H, s, ArOC*H*_3_), 2.42 (2H, q, *J* 7, ArC*H_2_*CH_3_), 1.07 (3H, t, *J* 7, ArCH_2_C*H_3_*); ^13^C NMR (75 MHz, CDCl_3_) δ; 192.8, 157.4, 143.4, 135.6, 134.8, 132.8, 131.1, 129.0, 128.7, 128.4, 125.6, 119.0, 116.0, 56.2, 26.7, 14.9; *m/z* (CI) 241 (M+1, 100%). Mass measurement 240.1154, C_16_H_16_O_2_ requires 240.1155.

*6-Methoxy-2’-isopropylbiphenyl-2-carbaldehyde* (**6f**)

In the same way, oxazoline **5f** (4.6 g, 14.2 mmol) gave the title compound **6f** as a white solid (1.6 g, 44%). Mp 70–72 °C; *R_f_* 0.75 (7:3 v/v petroleum ether/EtOAc); IR ν_max_ (thin film) (DCM) 3560, 2961, 2744, 1690 (C=O), 1592, 1467, 1385, 1261, 1065, 911 cm^-1^; ^1^H NMR (300 MHz, CDCl_3_) δ; 9.68 (1H, s, C*H*O), 7.68 (1H, d, *J* 7, Ar*H*), 7.53 (1H, d, *J* 8, Ar*H*), 7.46 (1H, t, *J* 5, Ar*H*), 7.32–7.20 (3H, m, Ar*H*), 7.11 (1H, d, *J* 8, Ar*H*), 3.80 (3H, s, ArOC*H*_3_), 2.65 (1H, sep, *J* 8, ArC*H*(CH_3_)_2_), 1.22 (3H, d, *J* 7 ArOCH(C*H_3_*)_2_) 1.07 (H, d, *J* 7 ArOCH(C*H_3_*)_2_); ^13^C NMR (75 MHz, CDCl_3_) δ; 192.7, 157.5, 148.3, 135.8, 135.0, 132.0, 131.0, 129.0, 125.7, 119.7, 119.1, 115.9, 115.1, 56.01, 30.8, 24.2, 23.7; *m/z* (CI) 255 (M+1, 70%). Mass measurement 254.1311, C_17_H_18_O_2_ requires 254.1307.

*3-Methoxy-2-naphthalen-1-ylbenzaldehyde* (**6g**)

In the same way, oxazoline **5g** (1.1 g, 3.3 mmol) gave the title compound **6g** as a white solid (270 mg, 31%). Mp 65–68 °C; *R_f_* 0.75 (7:3 v/v petroleum ether/EtOAc); IR ν_max_ (thin film) (DCM) 3040, 2960, 2930, 1683, 1261 cm^-1^; ^1^H NMR (300 MHz, CDCl_3_) δ; 9.4 (1H, s, C*H*O), 7.2–7.9 (10H, m, Ar*H*), 3.60 (3H, s, OCH_3_); ^13^C NMR (75 MHz, CDCl_3_) δ; 190.5 163.1, 138.5, 137.5, 137.1, 136.1, 130.1, 129.4, 129.3, 127.6, 127.3, 125.6, 124.7, 122.3, 120.1, 119.3, 53.1; (O*C*H_3_); *m/z* (CI) 263 (M+1, 100%). Mass measurement 262.0985, C_18_H_14_O_2_ requires 262.0994.

*(3R,7aS)-3-(6-Methoxy-2’-ethoxybiphenyl-2-yl)-2-phenylhexahydropyrrolo[1,2-c]imidazole* (**9b**)

Following the method described in the main paper, aldehyde **6b** (717 mg, 2.8 mmol) gave the title compound **9b** as a white solid (1.1 g, 93%). Mp 142–145 °C; ^1^H NMR spectra indicated a mixture of conformers in a 5:1ratio. Extensive purification by flash chromatography on alumina (eluent 5:1 v/v petroleum ether/EtOAc) resulted in the separation of one diastereoisomer (444 mg, 40% of initial yield). IR ν_max_ (thin film) (DCM) 3002, 2912, 1775, 1557, 1150, 875; ^1^H NMR (300 MHz, CDCl_3_) δ; 7.40–7.15 (7H, m, Ar*H*), 6.94 (2H, d, *J* 9 Ar*H*), 6.66 (2H, t, *J* 9, Ar*H*), 6.54 (1H, d, *J* 9, Ar*H*), 5.06 (1H, s, PhNC*H*N), 4.16 (2H, q, *J* 8, ArOC*H_2_*CH_3_), 3.76 (3H, s, O*CH_3_*) 3.23 (1H, m, NC*H*CH_2_), 2.62–2.58 (2H, m, NC*H_2_*), 2.28–2.20 (2H, m, NC*H_2_*), 1.86–1.77 (4H, m, NCH_2_C*H_2_*C*H_2_*), 1.23 (3H, *J* 7, ArOCH_2_C*H_3_*); ^13^C NMR (75 MHz, CDCl_3_) δ:158.0, 156.8, 146.3, 133.9, 132.9, 130.7, 129.1, 128.8, 128.6, 126.0, 122.3, 120.5, 118.9, 117.6, 116.3, 113.0, 112.2, 110.6, 81.6, 64.1, 63.9, 60.2, 56.3, 53.3, 28.4, 24.5, 15.2. Mass measurement *M+H^+^* 415.2376, C_27_H_30_N_2_O_2_ *M+H^+^* requires 415.2386. [α]_D_^25^ = 0.351 (*c* = 0.512, ethanol).

Also obtained was a mixture of both diastereoisomers (656 mg, 60% of initial yield).

*(3R,7aS)-3-(6-Methoxy-2’-isopropoxybiphenyl-2-yl)-2-phenylhexahydropyrrolo[1,2-c]imidazole* (**9c**)

In the same way, aldehyde **6c** (702 mg, 2.6 mmol) gave the title compound **9c** as a white solid (1.1 g, 96%). ^1^H NMR spectra indicated a mixture of conformers in a 5:1 ratio. Extensive purification by flash chromatography on alumina (eluent 5:1 v/v petroleum ether/EtOAc) resulted in the separation of one diastereoisomer (385 mg, 35% of initial yield) Mp 164–166 °C; *R_f_* 0.25 (10:1 v/v petroleum ether/EtOAc); IR ν_max_ (thin film) (DCM) 3622, 2057, 2832, 1680, 1597, 1504, 1368, 1258, 1125, 995; ^1^H NMR (300 MHz, CDCl_3_) δ; 7.40–7.02 (7H, m, Ar*H*), 6.96–6.88 (2H, m, Ar*H*), 6.66 (2H, t, *J* 8, Ar*H*), 6.52 (1H, d, *J* 8, Ar*H*), 5.05 (1H, s, PhNC*H*N), 4.08 (1H, m, ArOC*H*(CH_3_)_2_), 3.76 (3H, s, O*CH_3_*) 3.18 (1H, t, *J* 9, PhNCH_2_NC*H*), 2.74–2.66 (2H, m, NC*H_2_*), 2.28–2.20 (2H, m, NC*H_2_*), 1.84–1.60 (4H, m, NCH_2_C*H_2_*C*H_2_*), 0.86 (6H, d, *J* 8, ArOCH(C*H_3_*)*_2_*); ^13^C NMR (75 MHz, CDCl_3_) δ; 163.8, 157.4, 133.7, 131.5, 129.7, 128.6, 128.4, 128.1, 126.7, 126.1, 122.2, 121.7, 120.7, 120.4, 119.5, 116.1, 115.9, 114.1, 79.5, 76.9, 72.9, 69.3, 56.2, 28.3, 23.3, 22.6, 21.3; *m/z* (CI) 429 (M+1, 70%). Mass measurement 428.2445, C_28_H_32_N_2_O_2_ requires 428.2464. [α]_D_^25^ = −98.7 (*c* = 0.507, ethanol)

Also obtained was a mixture of both diastereoisomers (715 mg, 65% of initial yield).

*(3R,7aS)-3-(6-Methoxy-2’-methylbiphenyl-2-yl)-2-phenylhexahydropyrrolo[1,2-c]imidazole* (**9d**)

In the same way, aldehyde **6d** (142 mg, 0.62 mmol) gave the title compound **9d** as an off white oil (180 mg, 79%). ^1^H NMR spectra indicated a mixture of conformers in a 1:1 ratio. Extensive purification by flash chromatography on alumina (eluent 5:1 v/v petroleum ether/EtOAc) resulted in the separation of one diastereoisomer (72 mg, 40% of initial yield). IR ν_max_ (thin film) (DCM) 3012, 2933, 1637, 1599, 1050, 1434 cm^-1^; ^1^H NMR (300 MHz, CDCl_3_) δ; 7.65–7.00 (12H, m, Ar*H*), 4.96 (1H, s, PhNC*H*N), 3.60 (3H, s, O*CH_3_*), 3.12 (1H, m, PhNCH_2_NC*H*), 2.40 (2H, m, NC*H_2_*), 2.2–2.0 (5H, m, CH_3_ + NC*H_2_*), 1.80–1.75 (4H, m, NCH_2_C*H_2_*C*H_2_*); Mass measurement 384.2222, C_26_H_28_N_2_O requires 384.2202. [α]_D_^25^ = −198.75 (*c* = 0.107, ethanol).

Also obtained was a mixture of both diastereoisomers (108 mg, 60% of initial yield).

*(3R,7aS)-3-(6-Methoxy-2’-ethylbiphenyl-2-yl)-2-phenylhexahydropyrrolo[1,2-c]imidazole* (**9e**)

In the same way, aldehyde **6e** (744 mg, 3.1 mmol) gave the title compound **9e** as a yellow solid (1.2 g, 97%). ^1^H NMR spectra indicated a mixture of conformers in a 1:1 ratio. Extensive purification by flash chromatography on alumina (eluent 5:1 v/v petroleum ether/EtOAc) resulted in the separation of one diastereoisomer (300 mg, 25% of initial yield). Mp 142–144 °C; *R_f_* 0.20 (8:1 v/v petroleum ether/EtOAc); IR ν_max_ (thin film) (DCM) 3468, 2934, 2833, 1677 (impurity), 1598, 1466, 1437, 1366, 1257, 1205, 1136, 1069; ^1^H NMR (300 MHz, CDCl_3_) δ; 7.44–7.08 (6H, m, Ar*H*), 6.96–6.88 (2H, m, Ar*H*), 6.76–6.60 (2H, m, Ar*H*), 6.46 (1H, d, *J* 8, Ar*H*), 6.32 (1H, d, *J* 8, Ar*H*), 4.92 (1H, s, PhNC*H*N), 3.78 (3H, s, O*CH_3_*), 3.64 (1H, m, PhNCH_2_NC*H*), 2.58 (2H, q, *J* 7, ArC*H_2_*CH_3_), 2.48 (2H, m, NCH_2_), 2.20–2.10 (2H, m, NC*H_2_*), 1.84–1.70 (4H, m, NCH_2_C*H_2_*C*H_2_*), 1.18 (3H, t, *J* 7, ArCH_2_C*H_3_*); *m/z* (CI) 399 (M+1, 100%). Mass measurement *M+H^+^* 399.2428, C_27_H_30_N_2_O *M+H^+^* requires 399.2436. [α]_D_^25^ = −82.4 (*c* = 0.039, ethanol).

Also obtained was a mixture of both diastereoisomers (900 mg, 75% of initial yield).

*(3R,7aS)-3-(6-Methoxy-2’-isopropylbiphenyl-2-yl)-2-phenylhexahydropyrrolo[1,2-c]imidazole* (**9f**)

In the same way, aldehyde **6f** (812 mg, 3.2 mmol) gave the title compound **9f** as a white solid (1.3 g, 97%). Mp 142–145 °C; ^1^H NMR spectra indicated a mixture of diastereoisomers in a 1:1 ratio. Extensive purification by flash chromatography on alumina (eluent 5:1 v/v petroleum ether/EtOAc) resulted in the separation of one diastereoisomer (325 mg, 25% of initial yield) Mp 155–158 °C; *R_f_* 0.28 (5:1 v/v petroleum ether/EtOAc); IR ν_max_ (thin film) (DCM) 3344, 2959, 2834, 1734 (impurity), 1601, 1502, 1368, 1259, 1159, 895; ^1^H NMR (300 MHz, CDCl_3_) δ; 7.48–7.22 (5H, m, Ar*H*), 7.12 (2H, t, *J* 7, Ar*H*), 6.92 (2H, d, *J* 8, Ar*H*), 6.64 (1H, t, *J* 8, Ar*H*), 6.35 (2H, d, *J* 8, Ar*H*), 4.86 (1H, s, PhNC*H*N), 3.95 (1H, m, PhNCH_2_C*H*), 3.74 (3H, s, ArO*CH_3_*), 2.85 (1H, sep, *J* 7, ArC*H*(CH_3_)_2_), 2.4 (2H, m, NC*H_2_*), 2.0–2.2 (2H, m, NC*H_2_*), 1.80–1.75 (4H, m, NCH_2_C*H_2_*C*H_2_*), 1.37 (3H, d, *J* 6, ArCH(C*H*_3_)_2_), 1.26 (3H, d, *J* 6, ArCH(C*H*_3_)_2_); ^13^C NMR (75 MHz, CDCl_3_) δ; 163.8, 157.4, 146.9, 133.7, 131.5, 129.7, 128.6, 128.4, 128.3, 126.7, 122.2, 121.9, 121.5, 120.7, 120.3, 116.7, 115.2, 114.7, 79.5, 72.4, 71.9, 70.0, 67.2, 56.2, 28.3, 23.1, 22.3, 22.2; *m/z* (CI) 413 (M+1, 100%). Mass measurement *M+H* 413.2595, C_28_H_32_N_2_O *M+H* requires 413.2593. [α]_D_^25^ = −110.2 (*c* = 0.321, ethanol).

Also obtained was a mixture of both diastereoisomers (975 mg, 75% of initial yield).

*(3R,7aS)-3-(3-Methoxy-2-naphthalen-1-yl-phenyl)-2-phenylhexahydropyrrolo[1,2-c]imidazole* (**9g**)

In the same way, aldehyde **6g** (78 mg, 0.30 mmol) gave the title compound **9g** as a brown solid (101 mg, 83%). ^1^H NMR spectra indicated a mixture of diastereoisomers in a 1:1 ratio. Extensive purification by flash chromatography on alumina (eluent 5:1 v/v petroleum ether/EtOAc) resulted in the separation of one diastereoisomer (33 mg, 33% of initial yield). Mp 170–172 °C; *R_f_* 0.20 (5:1 v/v petroleum ether/EtOAc); IR ν_max_ (thin film) (DCM) 3012, 2933, 1637, 1599, 1050, 734 cm^-1^; ^1^H NMR (300 MHz, CDCl_3_) δ; 7.45–7.22 (15H, m, Ar*H*), 5.00 (1H, s, PhNC*H*N), 3.79 (3H, s, O*CH_3_*), 3.63–3.60 (1H, m, PhNCH_2_C*H*), 2.40 (2H, m, NC*H_2_*), 2.2–2.0 (2H, m, NC*H_2_*), 1.80–1.75 (4H, m, NCH_2_C*H_2_*C*H_2_*); *m/z* (CI) 401 (M+1, 100%). Mass measurement 420.2191, C_29_H_28_N_2_O requires 420.2202. [α]_D_^25^ = −12.6 (*c* = 0.044, ethanol).

Also obtained was a mixture of both diastereoisomers (67 mg, 67% of initial yield).

*(2S,4S,5R)-2-(6-Methoxy-2’-methylbiphenyl-2-yl)-3,5-dimethyl-4-phenyloxazolidine* (**10d**)

Following the method described in the main paper, **6d** (155 mg, 0.69 mmol) gave a crude product which was purified by flash chromatography on alumina (eluent 5:1 v/v petroleum ether/EtOAc) to yield the title compound **10d** as a white solid (181 mg, 71%). Mp 125–128 °C; ^1^H NMR spectra indicated a mixture of conformers at a ratio of 1:1. *R_f_* 0.38 (5:1 v/v petroleum ether/EtOAc); IR ν_max_ (thin film) (DCM) 3112, 2907, 1613, 1467, 1179, 731 cm^-1^; ^1^H NMR (300 MHz, CDCl_3_) δ; 7.75–7.05 (12H, m, Ar*H*), 5.05 (1H, d, *J* 7, PhC*H*) [other conformer at 4.82 (1H, d, *J* 4, PhC*H*)], 4.50 (1H, s, NC*H*O), [other conformer at 4.40 (1H, s, NC*H*O)], 3.75 (3H, s, OC*H_3_*), 2.85 (1H, qd, *J* 6, 4, CH_3_C*H*), 2.65 (3H, s, ArC*H_3_*), 2.10 (3H, s, NC*H_3_)*, 0.90 (3H, d, *J* 6, CHC*H_3_*); ^13^C NMR (75 MHz, CDCl_3_) δ; 162.0, 137.2, 137.0, 133.4, 128.8, 128.7, 128.0, 127.1, 126.9, 126.5, 125.8, 124.9, 124.0, 123.9, 123.5, 122.1, 114.3, 113.7, 92.1, 79.9, 63.7, 56.5, 35.9, 18.2, 14.5; *m/z* (CI) 374 (M+1, 100%), Mass measurement 373.2031, C_25_H_27_NO_2_ requires 373.2042.

*(2S,4S,5R)-2-(3-Methoxy-2-naphthalen-1-yl-phenyl)-3,4-dimethyl-5-phenyloxazolidine* (**10g**)

In the same way, from aldehyde **6g** (224 mg, 0.85 mmol), the crude product was purified by flash chromatography on alumina (eluent 5:1 v/v petroleum ether/EtOAc) to yield the title compound **10g** as a white solid (265 mg, 76%). Mp 165–168 °C; ^1^H NMR spectra indicated a mixture of conformers at a ratio of 1:1. *R_f_* 0.24 (5:1 v/v petroleum ether/EtOAc); IR ν_max_ (thin film) (DCM) 3084, 2832, 1712 (impurity), 1391, 1099, 735 cm^-1^; ^1^H NMR (300 MHz, CDCl_3_) δ; 7.65–7.23 (15H, m, Ar*H*), 4.7 (1H, d, *J* 6, PhC*H*), 4.25 (1H, s, NC*H*O) [other conformer at 4.12 (1H, s, NC*H*O)], 3.50 (3H, s, OC C*H_3_*), 2.85(1H, qn, *J* 6, CH_3_C*H*), 2.45 (3H, s, N C*H_3_*), 0.82 (3H, d, *J* 6, CHCC*H_3_*); ^13^C NMR (75 MHz, CDCl_3_) 158.3, 139.5, 135.6, 135.0, 134.3, 133.2, 130.2, 129.5, 129.2, 128.8, 128.5, 128.4, 127.9, 127.5, 126.9, 126.7, 126.5, 126.4, 122.1, 120.9, 117.6, 116.5, 91.2, 85.4, 60.3, 59.7, 35.5, 14.8; *m/z* (CI) 410 (M+1, 100%). Mass measurement 409.2031, C_28_H_27_NO_2_ requires 409.2042.

# General procedure for deprotection and reduction of imidazolidines 9 to yield alcohols 11

A solution of the imidazolidine **9** (1.0 mmol) was dissolved in THF (20 mL) and cooled to −5 °C under nitrogen. A solution of 1M HCl (2.5 equiv) and water (1.1 equiv) was added dropwise, and the solution was stirred for 35 min. Sodium borohydride (2.7 equiv) and sodium methoxide (15 equiv) were dissolved in methanol (10 mL) with a few droplets of ethanol. This was added dropwise to the reaction mixture. It was stirred at −5 °C for a further 30 min, then allowed to warm to room temperature over an hour. The resulting mixture was evaporated under reduced pressure (without applying any external heat), then extracted with diethyl ether, washed with water (3 x 30 mL), brine (3 x 30 mL), and the combined organic layers were dried over MgSO_4_, concentrated under reduced pressure (again without applying any external heat), to afford the crude alcohol, which was purified by flash chromatography.

*(P)-2,2’-Dimethoxy-6-hydroxymethylbiphenyl* (**11a**)

In the same way, imidazolidine **9a** (50 mg, 0.125 mmol) gave, after purification by flash chromatography (eluent 5:1 v/v petroleum ether/EtOAc), the title compound **11a** as a clear oil (18 mg, 55%). Integration of the ^1^H NMR spectrum in the presence of TFAE gave an enantiomeric ratio of >99:1. *R_f_* 0.4 (5:1 v/v petroleum ether/EtOAc); IR ν_max_ (thin film) (DCM) 3477, 1686, 1443, 1208, 845 cm^-1^; ^1^H NMR (300 MHz, CDCl_3_) δ; 7.46–7.05 (7H, m, Ar*H*), 4.40 (2H, bs, ArC*H_2_*OH), 3.80 (6H, s, ArOC*H_3_*); *m/z* (CI) 245 (M+1, 30%), 262 (M+18, 100%). Mass measurement 244.1096, C_15_H_16_O_3_ requires 244.1099.

*(P)-2-Methoxy-2’-isopropoxy-6-hydroxymethylbiphenyl* (**11c**)

In the same way, imidazolidine **9c** (100 mg, 0.23 mmol) gave the title compound **11c** as a yellow oil (18 mg, 28%). Integration of the ^1^H NMR spectrum in the presence of TFAE gave an enantiomeric ratio of >99:1. *R_f_* 0.42 (5:1 v/v petroleum ether/EtOAc); IR ν_max_ (thin film) (DCM) 3438–3387 (OH), 1680, 1440, 1205, 1137, 845, 803 cm^-1^; ^1^H NMR (300 MHz, CDCl_3_) δ; 7.40–7.32 (2H, m, Ar*H*), 7.20–7.12 (3H, m, Ar*H*), 7.09 (1H, t, *J* 8, Ar*H*), 6.96 (1H, d, *J* 8, Ar*H*), 4.30 (2H, bs, ArC*H_2_*OH), 4.24 (1H, m, ArOC*H*(CH_3_)_2_), 3.78 (3H, s, ArOC*H_3_*), 1.22 (3H, d, *J* 6, ArOCH(C*H_3_*)_2_), 1.01(3H, d, *J* 6, ArOCH(C*H_3_*)_2_); ^13^C NMR (75 MHz, CDCl_3_) δ; 157.3, 155.5, 141.2, 132.4, 129.0, 128.4, 127.9, 127.1, 122.1, 121.7, 117.8, 110.5, 73.3, 64.3, 56.0, 22.4, 22.0; *m/z* (CI) 290 (M+18, 60%). Mass measurement *M +* *NH_4_* 290.1751, C_17_H_20_O_3_ *M +* *NH_4_* requires 290.1751.

*(P)-2-Methoxy-2’-methyl-6-hydroxymethylbiphenyl* (**11d**)

In the same way, imidazolidine **9d** (100 mg, 0.25 mmol) gave the title compound **11d** as a clear oil (25 mg, 44%). Integration of the ^1^H NMR spectrum in the presence of TFAE gave an enantiomeric ratio of >99:1. *R_f_* 0.38 (5:1 v/v petroleum ether/EtOAc); IR ν_max_ (thin film) (DCM) 3330, 2955, 1467, 1440, 1261, 1207, 1134, 1044 cm^-1^; ^1^H NMR (300 MHz, CDCl_3_) δ; 7.42–6.85 (7H, m, Ar), 4.35 (2H, bs, ArC*H_2_*OH), 3.78 (3H, s, ArOC*H_3_*), 2.30 (3H, s, ArC*H_3_*); *m/z* (CI) 229 (M+1, 40%), 246 (M+18, 100%). Mass measurement 228.1153, C_15_H_16_O_2_ requires 228.1150.

*(P)-2-Methoxy-2’-ethyl-6-hydroxymethylbiphenyl* (**11e**)

In the same way, imidazolidine **9e** (75 mg, 0.19 mmol) gave the title compound **11e** as a yellow oil (18 mg, 43%). Integration of the ^1^H NMR spectrum in the presence of TFAE gave an enantiomeric ratio of >99:1. *R_f_* 0.40 (5:1 v/v petroleum ether/EtOAc); IR ν_max_ (thin film) (DCM) 3530–3331 (OH), 2964, 1579, 1467, 1356, 1263, 1135, 1045, 830, 801 cm^-1^; ^1^H NMR (300 MHz, CDCl_3_) δ; 7.40–2.28 (4H, m, Ar*H*), 7.22 (1H, d, *J* 8, Ar*H*), 7.12 (1H, d, *J* 8, Ar*H*), 6.96 (1H, d, *J* 8, Ar*H*), 4.38 (2H, bs, ArC*H_2_*OH), 3.78 (3H, s, ArOC*H_3_*), 2.37 (2H, q, *J* 7, ArC*H_2_*CH_3_), 1.08 (3H, t, *J* 7, ArCH_2_C*H_3_*); ^13^C NMR (75 MHz, CDCl_3_) δ; 157.8, 138.8, 136.2, 136.3, 130.1, 128.9, 128.1, 127.8, 126.2, 125.9, 120.0, 110.1, 63.2, 26.2, 15.2; *m/z* (CI) 243 (M+1, 35%), 260 (M+18, 100%). Mass measurement *M +* *NH_4_* 260.1650, C_16_H_18_O_2_ *M +* *NH_4_* requires 260.1651.

# Reference

[1] Meyers, A. I.; Flanagan, M. E. *Org. Synth.* **1993,** *71,* 107.
